# Supplementary figures and images for: Fly-QMA: Automated analysis of mosaic imaginal discs in Drosophila
Source: PLoS Comput Biol. 2020 Mar 3;16(3):e1007406. doi: 10.1371/journal.pcbi.1007406 (PMC7100978; doi:10.1371/journal.pcbi.1007406)

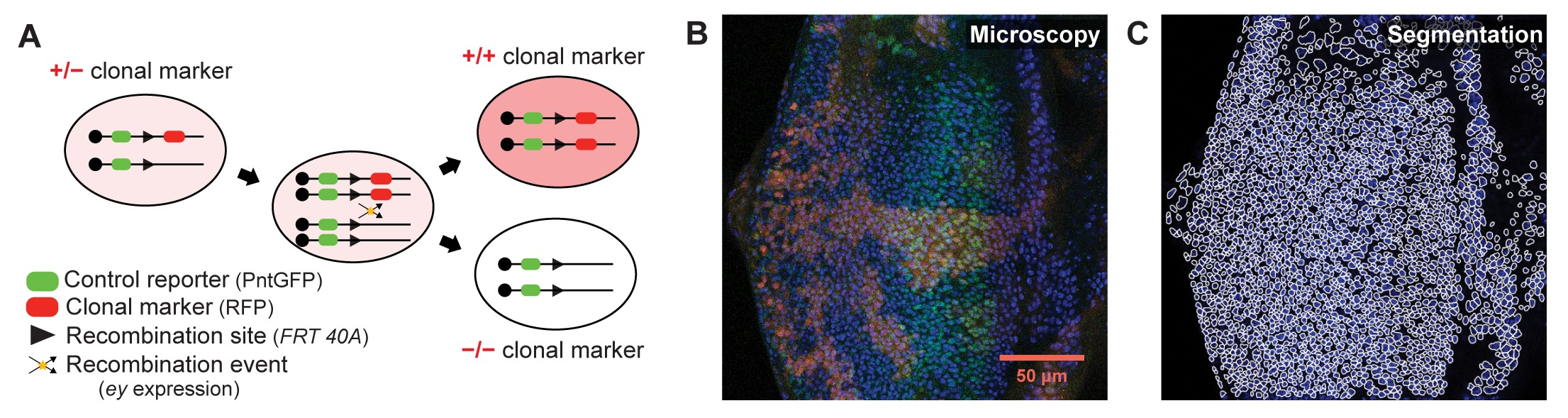

Supplement: S1 Fig — (A) Genetic schema for a bleedthrough control experiment. Red and green ovals represent genes encoding a RFP-tagged clonal marker and a GFP-tagged control reporter, respectively. Black lines depict a genomic locus. Recombination does not affect gene dosage of the control reporter, so GFP variation across clones is attributed to fluorescence bleedthrough. (B) Confocal image of an eye imaginal disc. Red, green, and blue reflect clonal marker, control reporter, and nuclear stain fluorescence, respectively. (C) Segmentation of the DAPI nuclear stain. White lines show individual segments. (TIF) [file pcbi.1007406.s001.tif]

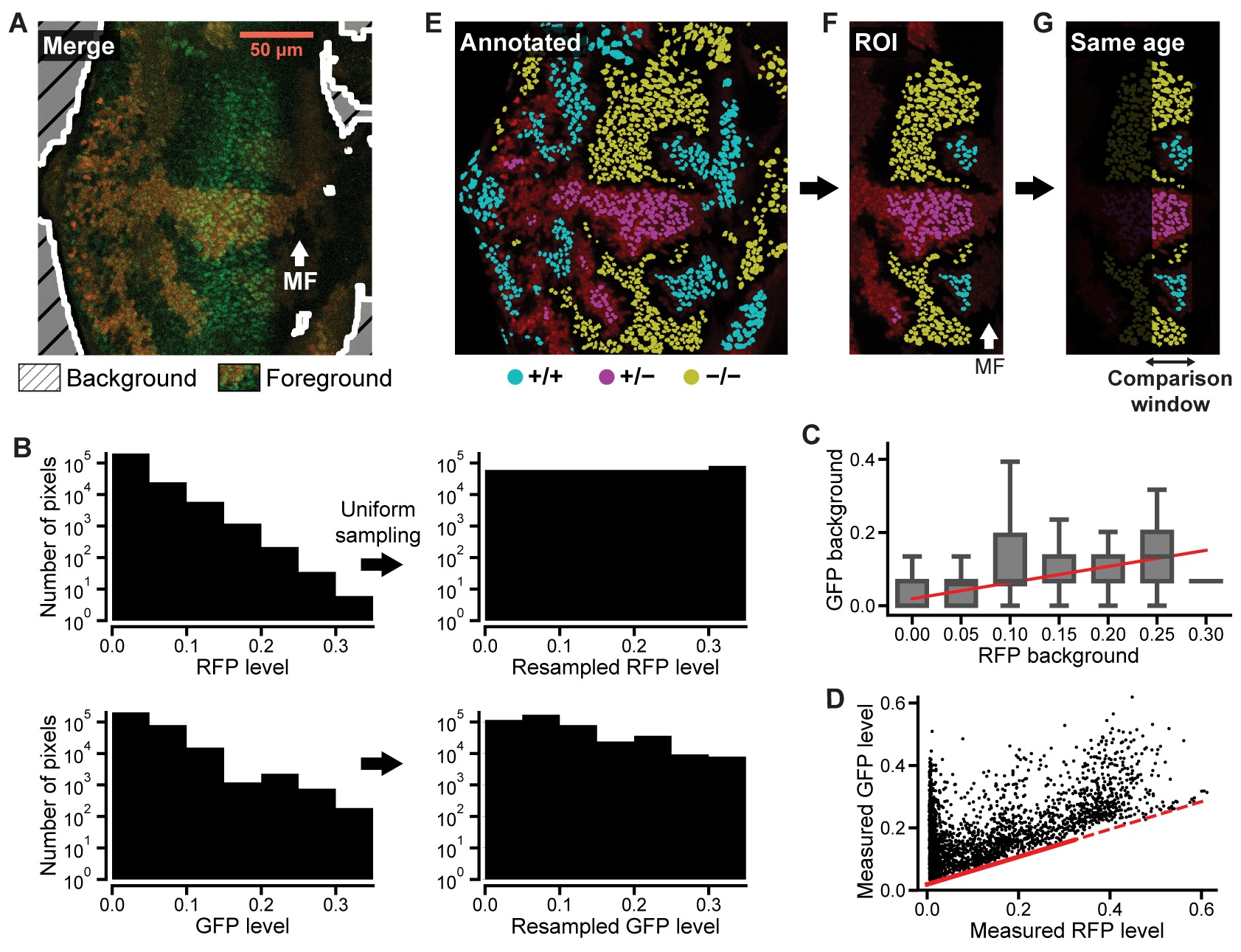

Supplement: S2 Fig — (A) Extraction of background pixels (striped region). Foreground includes the merged RFP and GFP images, surrounded by a white line. White arrow marks the morphogenetic furrow (MF). (B) Background pixel values are resampled such that RFP intensities are uniformly distributed. (C) A generalized linear model characterizes the contribution of RFP bleedthrough to GFP fluorescence. Boxes reflect windowed distributions of resampled background pixel intensities. Red line shows the model fit. (D) Measured GFP levels before bleedthrough correction. Markers represent individual nuclei. Red line shows the inferred contributions of RFP fluorescence bleedthrough. Dashed portion is extrapolated. (E-G) Data curation prior to statistical comparison of GFP levels. (E) Cells on the periphery of each clone are excluded. (F) The selection is limited to the region of elevated GFP expression near the MF. (G) It is further limited to cells of the same developmental age, defined by their relative positions along the x-axis. (TIF) [file pcbi.1007406.s002.tif]

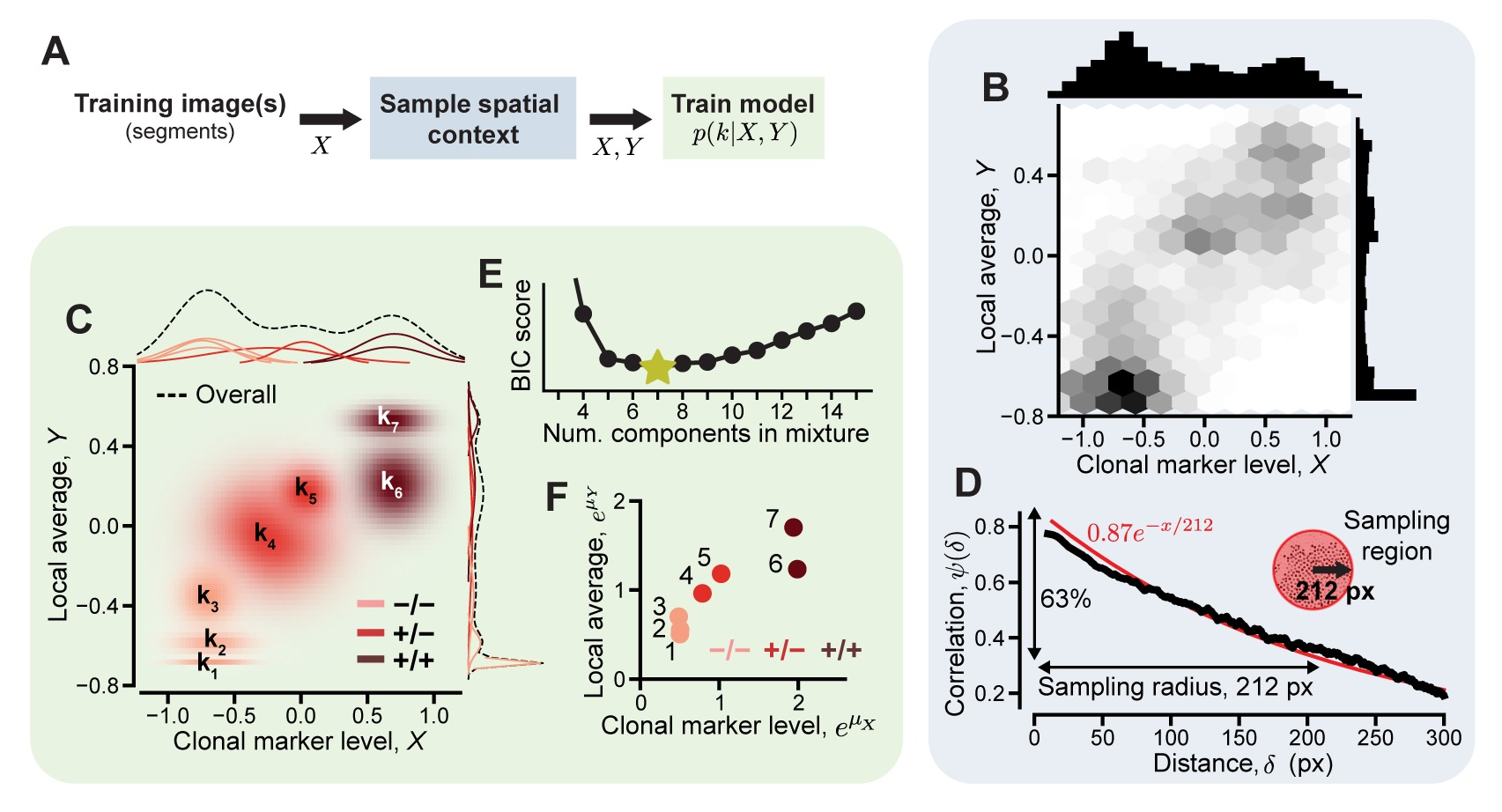

Supplement: S3 Fig — (A) One or more images are segmented, yielding a set of fluorescence measurements X. These are used to sample the spatial context Y of the neighborhood surrounding each cell. Both sets of values are used to train a mixture model. Subsequent panels demonstrate these procedures using the example shown in S3 Fig C. (B) Expression levels are jointly distributed with the local average among neighboring cells. Center panel shows the joint distribution. Top and right bar plots show marginal distributions. (C) Mixture model identifies seven distinct components ki. Center panel shows position and spread of each component. Top and right panels show marginal components scaled by their respective weights. Red shading denotes the label mi assigned to each component. The model predicts the posterior probabilities that a given sample (X, Y) belongs to each component. (D) Neighborhood size is estimated by computing the decay constant of the spatial correlation function, ψ(δ). Black line shows the moving average of ψ(δ), red line shows an exponential fit. Inset shows the resultant sampling region. (E) The optimal number of mixture components is determined by minimizing BIC score. (F) Mixture components are labeled by k-means clustering their mean values. Markers reflect the component means, colors denote the assigned label. (TIF) [file pcbi.1007406.s003.tif]

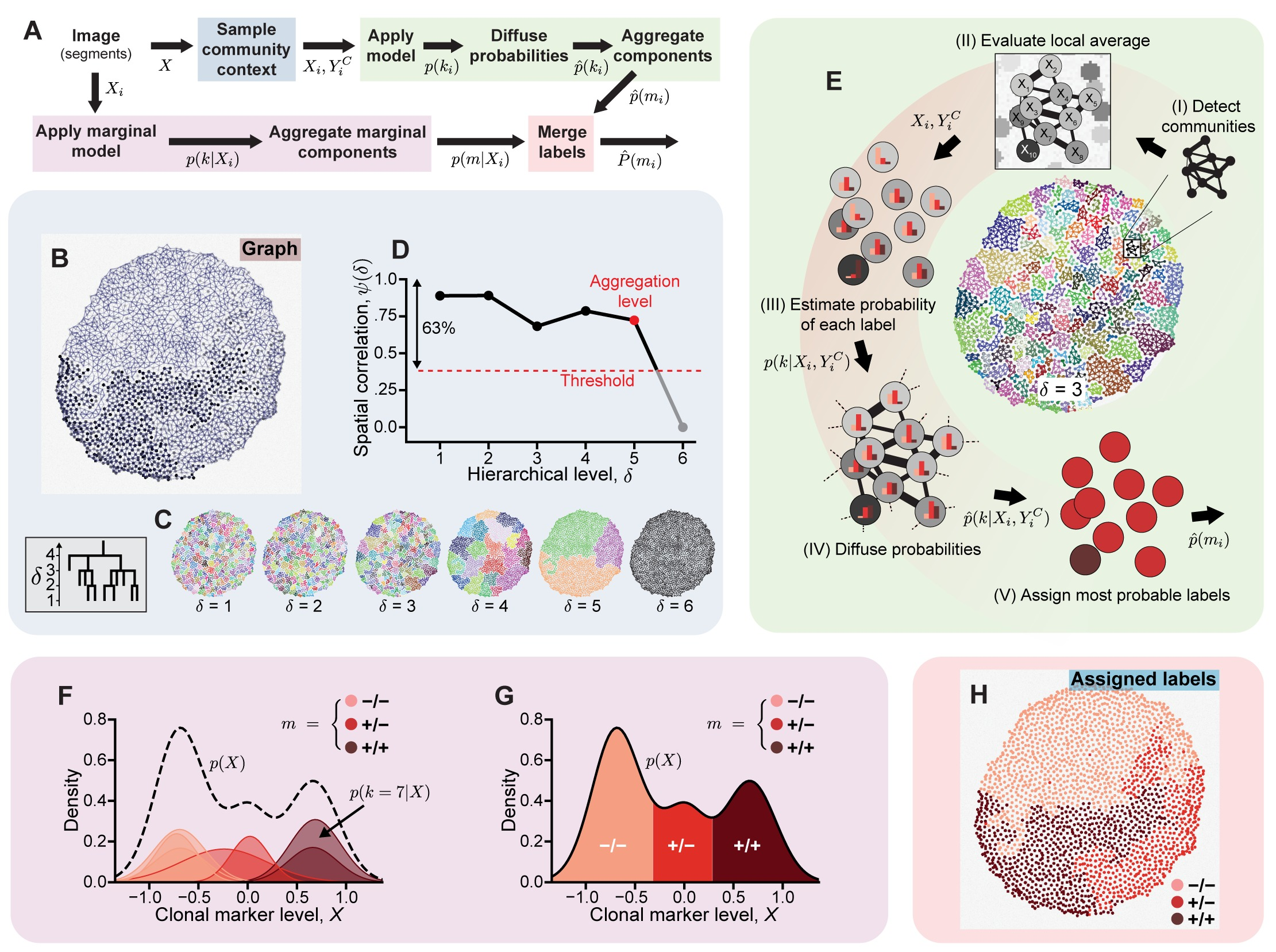

Supplement: S4 Fig — (A) Measurements are used to sample spatial contexts before the trained model is applied (blue and green path). In parallel, measurements are labeled using a marginal projection of the trained model (magenta path). The labels are then merged (red path). (B-D) Spatial context sampling. (B) Weighted undirected graph connecting adjacent cells. Line width reflects expression similarity between neighbors. (C) Community resolution is defined by aggregating clusters that fall below a hierarchical cut level δ. Panels show increasing levels of aggregation. Colors denote distinct communities. (D) Cut level is chosen by finding the maximum level (red dot) that remains lower than the decay constant of the spatial correlation function, ψ(δ) (black line). Panel E depicts aggregation below the third level for ease of visualization. (E) Application of the mixture model. (I) The graph contains distinct communities of locally similar expression. (II) Mean expression level within each community serves as the local average for each cell. (III) Mixture model estimates the probability that each cell belongs to each of its component. Bar plots within each cell illustrate the cumulative probability of each label. (IV) Posterior probabilities are diffused across the graph. (V) Each cell is assigned the most probable label. (F,G) Application of a marginal mixture model. (F) Marginal mixture components, shaded by their mapped labels. Dashed line is the overall marginal density. (G) Marginal classifier labels cells strictly on the basis of their individual fluorescence level. Red shading denotes the most probable label for each level. (H) Annotated measurements. Red shading denotes the assigned label. Labels with low confidence P^(mi)<0.8 are replaced by their marginal counterparts. (TIF) [file pcbi.1007406.s004.tif]

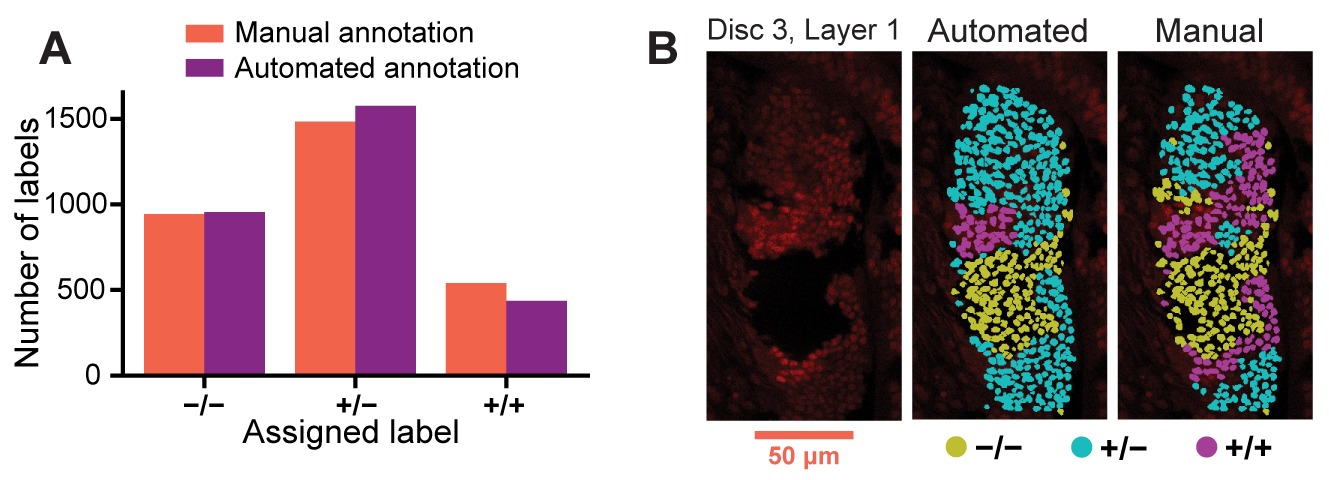

Supplement: S5 Fig — (A) Distribution of labels among each possible value. (B) Visual comparison of the sole instance in which automated and manual annotation differ. Image shows clonal marker fluorescence, colors denote the assigned label. (TIF) [file pcbi.1007406.s005.tif]

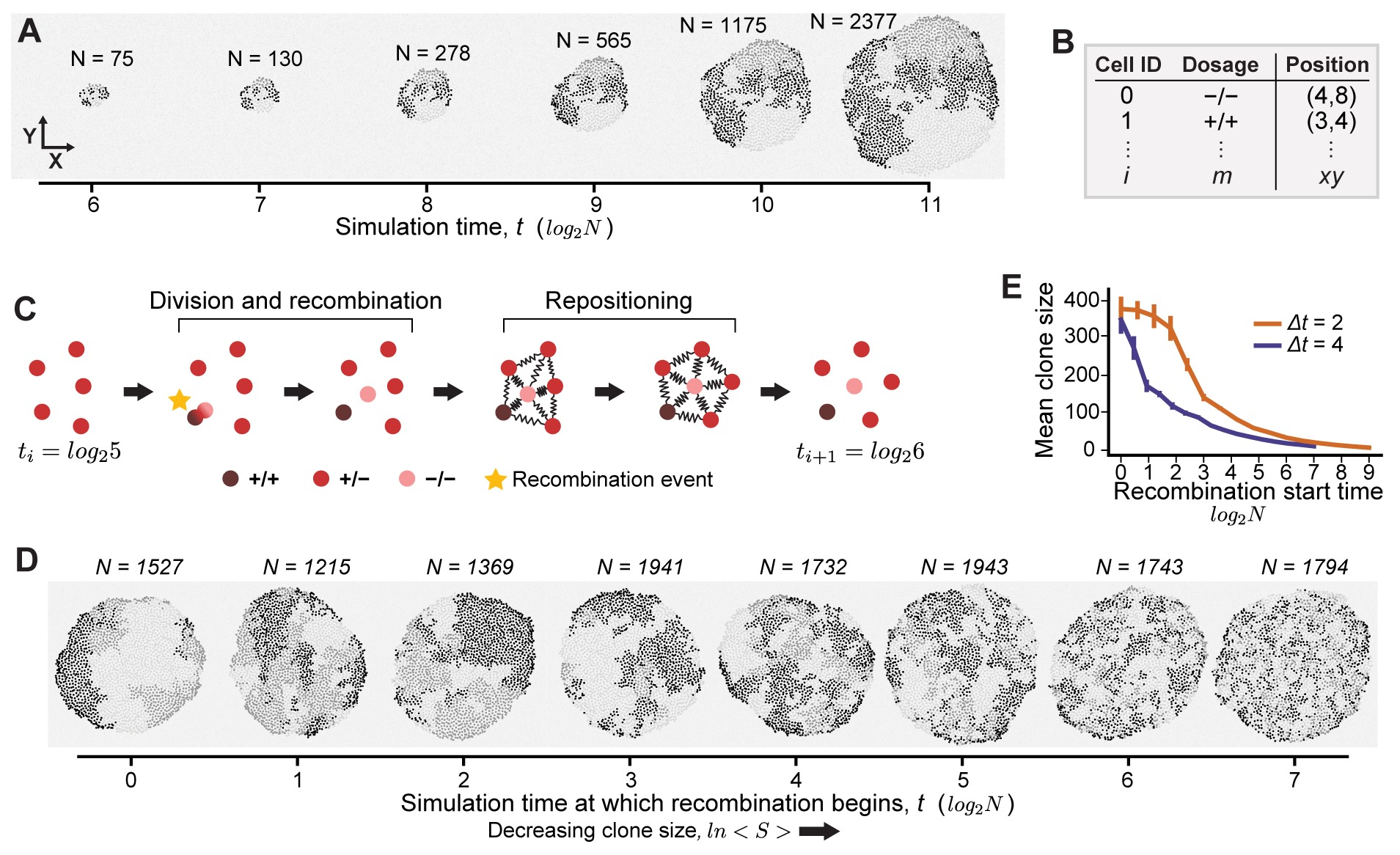

Supplement: S6 Fig — (A) Partial simulation time course. Each marker depicts a cell. Greyscale intensity reflects clonal marker gene dosage. Simulation time reflects the approximate number of cell divisions since the initial seed. (B) Simulations yield gene dosages and spatial coordinates for each cell. (C) Single iteration of an example simulation. Circles represent individual cells, red shading denotes clonal marker dosage. Cycles of cell division, recombination, and repositioning are repeated until the simulation reaches a specified end time (t > 11 in panel A). (D) Cultures simulated with varying recombination start times. All cultures were subject to four generations of recombination (δt = 4). Recombination start time increases from left to right. Later recombination events generally yield smaller clones. (E) Mean clone size (cells per clone) as a function of the recombination start time. Colors denote recombination period duration. Error bars reflect standard error of the mean across 50 replicates. Clone size generally decreases as recombination is limited to later times. (TIF) [file pcbi.1007406.s006.tif]

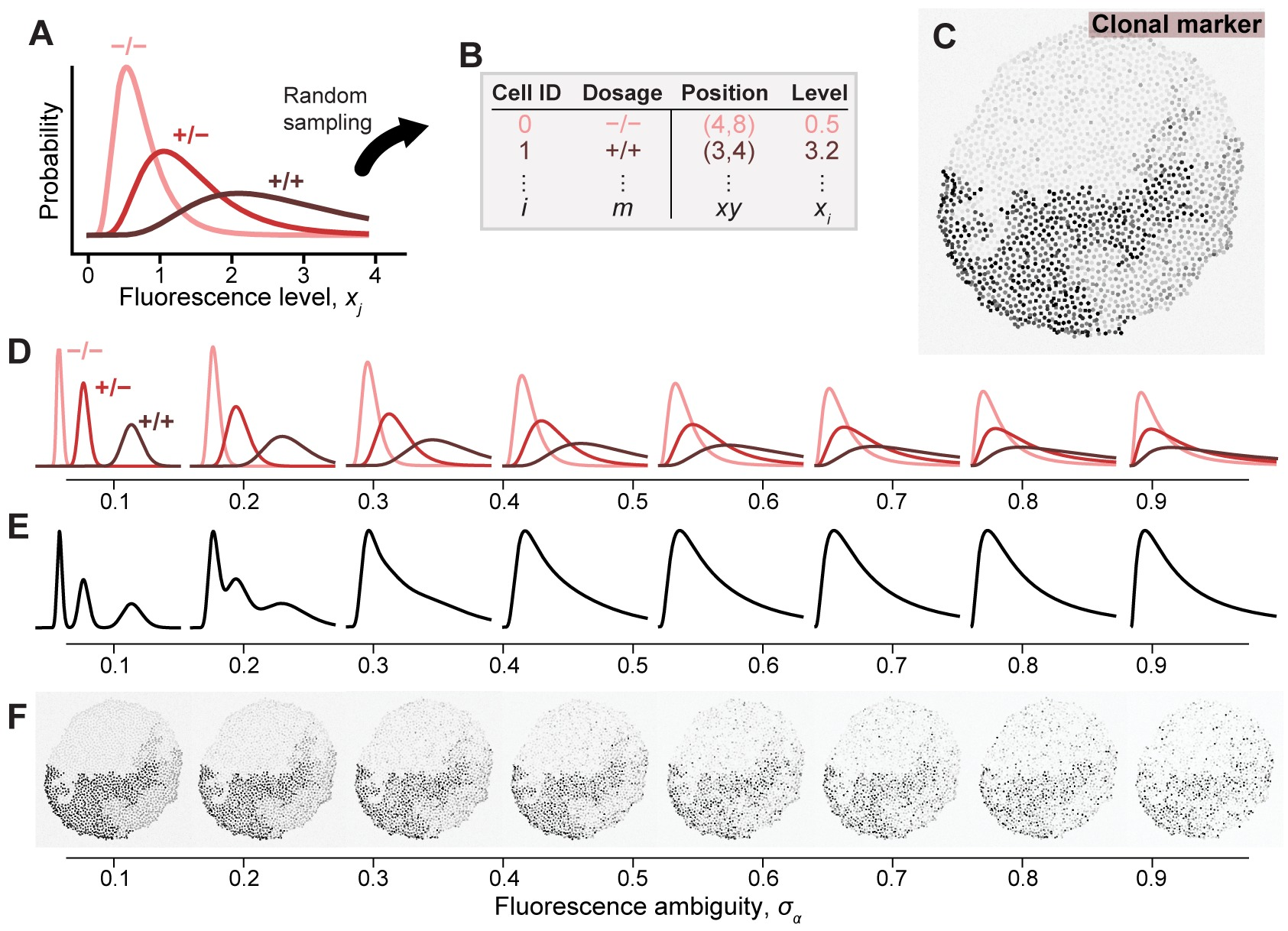

Supplement: S7 Fig — (A) Fluorescence levels are sampled from lognormal distributions conditioned upon gene dosage. (B) Synthetic data include a measured fluorescence level for each reporter in each cell. Text color reflects the generative distribution in A. (C) Synthetic image of clonal marker fluorescence when σα = 0.25. Each nucleus is shaded in accordance with its sampled fluorescence intensity. (D-F) Left to right, increasing the fluorescence ambiguity parameter broadens the overlap in fluorescence levels across gene dosages. (D) Distributions used to generate clonal marker fluorescence levels. Red shading denotes gene dosage. (E) Evenly weighted sum of the generative distributions. (F) Example images of clonal marker fluorescence. (TIF) [file pcbi.1007406.s007.tif]

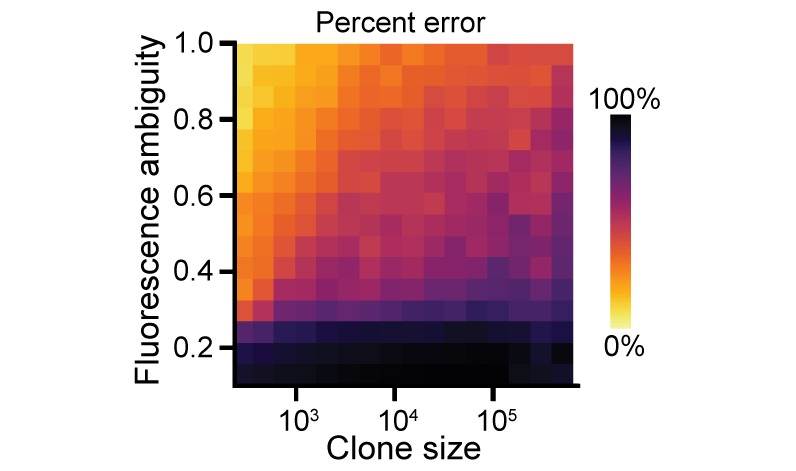

Supplement: S8 Fig — Each pixel reflects the average across 50 replicates. Clone size reflects the mean number of cells per clone. Performance improves with increasing clone size and worsens with increasing fluorescence ambiguity. (TIF) [file pcbi.1007406.s008.tif]

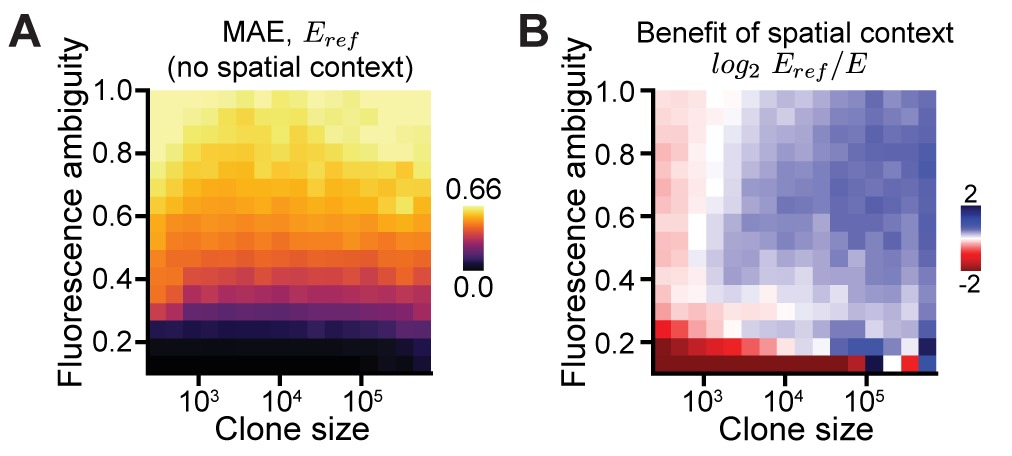

Supplement: S9 Fig — (A) MAE of labels assigned using a marginal classifier that neglects spatial context. Performance worsens with increasing fluorescence ambiguity but does not depend upon clone size. (B) Annotation performance relative to the marginal classifier. Color scale reflects the log2 fold-change in MAE when spatial context is neglected. Blue indicates that spatial context improves performance. (TIF) [file pcbi.1007406.s009.tif]
