# Supplementary material for: Fly-QMA: Automated analysis of mosaic imaginal discs in Drosophila
Source: PLoS Comput Biol. 2020 Mar 3;16(3):e1007406. doi: 10.1371/journal.pcbi.1007406 (PMC7100978; doi:10.1371/journal.pcbi.1007406)
Supplement: Attachment — Submitted filename: responses_to_reviewers.pdf [file pcbi.1007406.s010.pdf]

Responses to Reviewers:

We thank all of the reviewers for their insightful comments. Below we have provided responses to each of the concerns raised, making note of any corresponding revisions made to the manuscript or figures. We have preserved the original reviewer comments in black font, with our responses in blue.

Responses to Reviewer #1:

- Operating this algorithm seems complicated for someone who is not savvy with computer programming –even installing requires Python 3.6+. Even the tutorial seems complicated. Although some will be able to use it as is, it would be even more useful if the algorithm could be incorporated into a user-friendly and widely used program like Fiji/Image J.

We agree with the reviewer that the current implementation of the algorithms developed in this study is limited to those with programming experience. We are working on incorporating these methods into FlyEye Silhouette, our desktop application for Mac OS, which is easily installed (for free) via the Mac App Store and operated using a user-friendly graphical user interface that should be accessible to all users. We anticipate that it will take quite some time to develop and deploy these features, and so we have provided a fully functional python implementation in the meantime. We believe this approach is common within the field, and we have had success with it in the past. While our current desktop application was publicly released in 2019, the algorithms supporting it were first published as python scripts accompanying Pelaez et. al. (2015).

- Requires use of a 3D confocal stack, which is fine for some analyses but can it also be used for single images from epifluorescence scope (e.g., +GFP vs -GFP)?

While the current implementation supports the analysis of 3D confocal stacks, the algorithms do not utilize any 3D information. The "layer" dimensions is therefore not required, and both the algorithm and corresponding python implementation are well-suited to analyzing single images.

- Figure S8, "Fraction of nuclei correctly labeled during synthetic benchmarking" appears to be missing from the manuscript (the image labelled S8 appears to be S9, according to the legend information).

We thank the reviewer for bringing this to our attention, and we have included the missing figure (S9) in the revised submission.

Responses to Reviewer #2:

- The author summary partly contains exact replications of the abstract. This should be avoided and at least a slight rephrasing should be performed.

We have made a number of minor revisions to the author summary in order to further distance it from the abstract. The overall tone and content remain similar.

- The notation with <>-brackets for the average across all pixels seems unusual to me. Maybe rather use a horizontal bar on top of the variable letter?

This appears to vary by discipline. We were accustomed to seeing brackets but acknowledge that the bar notation is also common. Out of curiosity we looked it up and per ISO 80000-2 2019 both notations are equally valid, though apparently brackets specifically denote the arithmetic mean. We have left the brackets for the time being, but are open to switching to the bar notation if need be.

- To better understand that manual assessment is infeasible if the data set sizes grow, it would be good to have a comparison of the „processing times" of a human expert and the software.

The time required to manually label nuclei will depend on the tissue sample, image quality, software used, and the experience of the user. In our experience, using FlyEye Silhouette it takes roughly one to two hours to manually label the clonal marker phenotype of nuclei within a single layer of the image field. In practice, users may wish to annotate several layers from many tissue samples collected under multiple sets of conditions. The annotation algorithm achieves comparable accuracy in a matter of seconds. We have revised the manual annotation subsection to explicitly mention this speedup.

- If I understood it correctly, the simulated benchmark does not involve varying contrast in the different image regions. An easy addition for increased realism would be to add global illumination artifacts to the simulated images in order to also assess the validity of the CLAHE approach and to see if it works as expected for the simulated scenario.

The reviewer is correct that the simulated benchmark does not involve varying contrast within different regions of a single image. We want to emphasize that fluorescence levels within each nucleus are only ever quantified using the raw pixel intensities. The CLAHE approach is only used to facilitate segmentation of the nuclear stain, so the CLAHE operation does not affect the annotation step beyond simply identifying nuclei within the image. The reviewer raises the idea that CLAHE could also be used to resolve global illumination artifacts during the annotation step. This is a very interesting idea, but we have not attempted it as we find that the current algorithm provides adequate performance in all cases surveyed.

- The reference list mixes sentence case and title case for the titles and journal/conference names. While being a minor issue, I would recommend to use either one or the other but not mixing them.

We thank the reviewer for pointing this out. We have revised all titles to sentence case, and all journal/conference names to title case.

- Page 10, Fig. 2C should probably be Fig. 3C instead?

We again thank the reviewer – this should have read Fig. 3C and has been revised accordingly.

Responses to Reviewer #3:

- Whilst bleed through correction might be a useful feature it would be pertinent to eliminate bleed through at the image acquisition stage by careful choice of excitation and emission filters or tuning of detectors. It is not possible to judge from the data presented what the cause of the bleed through is because details of the image acquisition are not included in the methods. The authors should add this detail. I would expect to see it here in this case even though it is previously published.

We agree with the reviewer that bleed through should ideally be minimized or avoided during experimental design, through careful selection of fluorophore combinations. However, it is not always possible to choose combinations with mutually exclusive excitation-emission spectra, and/or to choose a set of tunable detectors that collect any wavelength range at will. For example, in our case we used an SP5 Leica confocal, with a tunable detector set to collect photons around the 460, 508, and 610 nm emission peaks of Dapi, GFP, and mRFP, respectively. We used the 405, 488, and 561 laser lines only collecting photons in the intervals 437-381, 491–555, and 570-644 nm for DAPI, GFP, and mRFP, respectively. However, we only had available an mRFP-NLS with FRT sites in the same chromosome to label the WT and heterozygous tissue in eye discs, and thus distinguish them from cells carrying *yan* mutant alleles. Before the mRFP molecules fully mature and emit predominantly around 610 nm, a fraction of them transits through a green-emitting immature state that partially overlaps with that of the GFP tagging Pointed. Therefore, some bleed through is expected when mRFP-NLS and Pnt-GFP are co-expressed, due to the partial overlap of the GFP emission spectrum with that of the immature mRFP protein. We refer the reviewer to Piatkevich et al (2010) - *Russian Chemical Reviews* 79(3) 243 - 258, which extensively discuses red fluorescent proteins and their properties. As bleed through is expected under some experimental conditions, our platform takes these scenarios into consideration and provides a quantitative approach to measure and discern its contribution. As suggested by the reviewer, we have added the following text to the Materials and methods section of the manuscript in order to detail the staining procedure and parameters used during image acquisition: *"Genetics, fly lines, immunohistochemistry, and imaging conditions related to this dataset have already been published (Bernasek, 2018). All discs were dissected in PBS, fixed in 4% paraformaldehyde for 30 min at room temperature, and permeabilized with PBS-Triton X-100 0.1% for 20 min at room temperature to allow DAPI penetration without perturbing the fluorescence of the Pnt-GFP protein. Discs were subsequently stained with a 4',6-diamidino-2-phenylindole (DAPI) nuclear marker, rinsed twice with PBS-Tween 0.5%, and mounted on Vecta Shield (Vector labs). Images were acquired using a Leica SP5 confocal equipped with a tunable detector. The 405, 488, and 561 nm lasers were used to excite DAPI, Pnt-GFP, and Ubi-mRFPnls, while photons were collected in the 437-481, 491–555, and 570-644 nm intervals for DAPI, GFP, and mRFP, respectively. Images were recorded with 16-bit resolution using a 40X oil objective. Discs were oriented with the dorso-ventral equator parallel to the horizontal axis, and all images captured at least six rows of ommatidia on either side of the equator. All discs were fixed, mounted, and imaged in parallel in order to reduce measurement error."*

- Historically the main bottleneck in automated clonal analyses (especially of data from fluorescent reporters) is in segmentation. This is often best achieved by trial and error because of the large variability between datasets and in their acquisition parameters. The authors are wise to point out that the pipeline can accommodate images segmented before hand but give no guidance on what the requirement of their software might be? Do they require the segmentation masks or regions of interest? Where is the standardised file structure described is it on github? Please clarify?

Usage guidelines for the software are generally minimal within the manuscript, as we aimed to focus on the algorithm rather than its implementation. Of course, practical details will ultimately be important to users, so we aim to address them on the associated github page.

To specifically answer the question raised by the reviewer, the python implementation of Fly-QMA reads segmentation masks as 2D numpy arrays of integers in which each element represents a single pixel within a 2D image. The integer value denotes the unique identity of each pixel, with some specified default value (e.g. 0 or -1) for background. In our experience, this is a common output format used by other segmentation platforms (such as skimage). Regions of interest use a similar format, but the array contains Boolean values (include vs exclude) for each pixel. These arrays would have to be manually added in accordance with the standardized file structure used by Fly-QMA, which is outlined under the 'Measuring Expression' subsection of the 'Getting Started' page on github (https://sebastianbernasek.github.io/flyqma/start.html). We thank the reviewer for exposing these gaps in the online documentation, and we are working to improve it accordingly.

- None of the microscopy images in any of the figures include scale bars and it is not clear what objective was used for image capture so it is hard to tell what a comparable system for image acquisition would be. Please revise all the figures to include scale bars.

We thank the reviewer for pointing this out, and have added the appropriate scale bars to all figures containing microscope images. The example images in the original manuscript were 228 µm x 228 µm, aside from panels C-H in Fig 2 which were cropped to a much smaller region.

- The analysis appears to be limited to nuclear signals as this segmentation occurs on nuclei. This is a limitation as often the analysis is designed to identify contiguous clones for which the extent of the cytoplasm may be important. This should be discussed.

The reviewer is correct that our analysis is limited to discrete fluorescence signals, though the analysis strategies (excluding segmentation) are not inherently limited to cell nuclei. Given a cell membrane segmentation mask, cytoplasmic reporter levels could be quantified and analyzed using our approach. However, we agree that our approach would not be well suited to purely contiguous clones in which adjacent cells cannot be distinguished. We have added the following text to the conclusions section of the manuscript to address this limitation: *"A notable limitation of our approach is its reliance upon reporter fluorescence*

levels within distinct cells or nuclei. This requirement for discrete measurements precludes analysis of contiguous clones in which cytosplasmic fluorescence signals are indistinguishable between adjacent cells. Our framework is thus well suited to many different mosaic analysis platforms deployed in imaginal discs, so long as reporter fluorescence levels are measured on a discrete basis."

We suspect that in principle our annotation algorithm could also be applied on a per-pixel rather than a per-nuclear signal basis, and could therefore be used to perform unsupervised clustering on any sort of image. However, we have not successfully annotated any images in such a manner because we found the task to be computationally infeasible, and we feel that the extensive code optimization required to test this approach falls beyond the scope of this study.

- I don't see is any attempt to derive the size (spatially or by number of cells) of the individual clones? Or to correct for the probability of multiple clones being adjacent to one another?

In general, we chose to focus on assaying gene expression (via reporter fluorescence) rather than the morphological properties of clones, but the latter are still accessible via Fly-QMA. We can estimate the number of cells per clone by evaluating the size of each connected component on the graph shown in Fig S4H. The physical area occupied by each identified clone can be estimated by summing up the area of the corresponding Delaunay triangulation (Fig S4B). This returns a value with units of squared pixels, which may then be converted to units of distance given some knowledge of the spatial resolution of the image. This clone size estimate is already available within Fly-QMA, but we have not implemented a scheme to estimate the number of coherent clones by correcting for the effects of clone aggregation as we understand this to be quite complicated for two-dimensional mosaics. We are aware of some approaches in the literature, namely limiting the analysis to imbalanced populations of clones and using a mean patch size correction factor empirically derived from geometric simulations (e.g. West, 1976). We feel that this method falls beyond the scope of the analyses presented here, but users could apply their own strategy using the mean patch sizes measured by Fly-QMA if they so choose.

West JD, Patches in the livers of chimaeric mice. Journal of Embryology and Experimental Morphology. Vol. 36, 1, pp. 151-161, 1976.

- Size is a key parameter and this is a common problem in many clonal analyses. Does the variation in intensities between clones with the same copy number mean that the algorithm can discriminate between adjacent clones? This seems unlikely. Therefore can the clone sizes be corrected for the probability of being multiple clones? Or, with the spatial information available, can large polyclonal units be identified in the annotation?

We agree with the reviewer that the algorithm offers no means of discriminating between adjacent clones of the same type, and therefore cannot discern the exact number of coherent clones in any given tissue. In the absence of any other discriminating features

between spatially adjacent clones of the same genotype (such as a second recombinant clonal marker) we are unsure how large polyclonal units could be reliably distinguished from single coherent clones.

- The authors don't do the excellent resources provided on github justice - please make it clear that there are tutorials and test data available there.

We appreciate the reviewer's positive feedback and have added the following sentences to the "Data and Software Availability" section of the revised manuscript: *"The associated code repository contains resources designed to help users analyze their own microscope images. These include code documentation, a guide to getting started with Fly-QMA, and an interactive tutorial that uses example data to demonstrate the core features of the software."*

We also added the following to the end of the introduction: *"Fly-QMA is freely available online (see Data and software availability), along with an interactive coding tutorial designed to acquaint users with the core software features by applying them to example data."*
